# COVID-19 Psychological Implications: The Role of Shame and Guilt

**DOI:** 10.3389/fpsyg.2020.571828

**Published:** 2020-11-03

**Authors:** Cesare Cavalera

**Affiliations:** Department of Psychology, Catholic University of Milan, Milan, Italy

**Keywords:** COVID-19, shame, guilt, trauma, mental disorders

The recent spread of COVID-19 may have dramatic consequences for mental health. As the present pandemic may imply an exposure to death or threatened death or serious physical injury, this situation involves a prolonged alert condition that can have significant psychological consequences on several levels (Greenberg et al., 2020). Besides the constant fear of being infected, other negative emotions may represent a serious issue for mental health. Shame and guilt are two distinct negative self-conscious emotions that can be triggered by the present pandemic. As evidenced by Tangney and colleagues (Tangney and Dearing, 2002; Tracy et al., 2007) and by recent contributions (Cândea and Szentagotai-Tǎta, 2018), when shame and guilt are not properly recognized and managed, they are in various ways related with severe psychological symptoms that can become a serious threat for mental health. In the present paper, shame and guilt functioning is presented, and their relations with the current pandemic situation are discussed.

## Am I Infecting Others? Guilt Implications

The COVID-19 pandemic may be serious not only in terms of the ongoing threat it poses to personal safety but also in terms of the intrusive experience of guilt, which may undermine mental well-being.

The emotion of guilt involves self-criticism for a specific action and the harm it may cause others. It is linked to a concern with one's effect on others and to empathy by taking their perspective (Tangney and Dearing, 2002). Therefore, in many cases, guilt can be considered adaptive and constructive because it usually prompts proactive actions, like confessing and apologizing, bringing benefits to interpersonal relationships as a result. Nevertheless, guilt can also be maladaptive when it becomes “free-floating,” unrelated to specific contexts (Cândea and Szentagotai-Tǎta, 2018). Guilt can become maladaptive for mental health when individuals develop an exaggerated sense of responsibility for events that occur out of their control, or when reparation is not possible for a behavior (Leary and Tangney, 2011; Cherry et al., 2017). Untreated guilt that is related with a stressor event can be associated with severe mental health complications including posttraumatic stress disorder, depression, suicidal ideation, problematic substance use, and poorer functioning and quality of life (Tracy et al., 2007; Bryan et al., 2014; Browne et al., 2015; Griffin et al., 2019). Also, in COVID-19 stress-related events, maladaptive guilt can result when distress is taken as evidence of wrongdoing (e.g., “I feel terrible so I must have done something horribly wrong”) and making it impossible to evaluate actual evidence and come to an accurate perspective or change one's behavior, thereby maintaining and worsening personal distress (Haller et al., 2020; Sahoo et al., 2020). This type of emotion involves feelings of personal hyper-responsibility over aspects that the individual has little or no control. Therefore, guilt can be easily elicited by experiences related to COVID-19 transmission (Haller et al., 2020; Ransing et al., 2020). A prolonged condition of uncertainty and constant alert related to COVID-19 combined with a fear of infecting others can determine a threat of maladaptive guilt that has disruptive consequences for mental health (Brooks et al., 2020). This may be true not only for health care workers but for all people who cannot adopt smart work solutions. Even where all the necessary safety measures are followed carefully, recurrent thoughts may be related to the possibility of being a COVID-19 carrier and a risk to family members.

This emotional experience may be amplified among the caregivers, family members, friends, or coworkers of people who have actually contracted COVID-19, or worse, died from it. Given that in many cases the source of COVID-19 cannot be easily traced and anyone can potentially be an asymptomatic carrier, recursive thoughts related to a real, or supposed personal responsibility of infection may prove oppressive. These elements expose everyone who has been in contact with people who have been infected with or died from COVID-19 to a constant situation of uncertainty, which may elicit maladaptive guilt related to inappropriate or exaggerated feelings of responsibility (Cândea and Szentagotai-Tǎta, 2018). Moreover, bans on people coming into close contact with others who are dying from COVID-19 may have traumatic implications that prevent family members and caregivers from progressing through the grieving process.

Guilt, as an emotional experience, may also prove a pertinent issue for people actually infected with COVID-19 (Ransing et al., 2020). As treatment implies strict contagion control measures, even a mild infection often requires a forced quarantine that imposes social distancing and a sudden change of domestic habits (Brooks et al., 2020). It is important here to underline the negative effects of the quarantine on the emotional well-being. In this situation, feelings of hyper-responsibility for ruining one's own life and those of other family members may arise. Such emotions may be particularly disruptive when the infection shows clinical complications that require hospitalization (Brooks et al., 2020): the condition of loneliness combined with the fear of dying may amplify intrusive thoughts of guilt in the patient (e.g. “I didn't pay enough attention,” “I made a bad mistake”). Narrative lived experiences reported by Sahoo et al. (2020) evidenced that feelings of guilt of infecting near and dear ones adds on to the pain of remaining social isolated from the family in a “locked up” state that can have disruptive consequences on emotional reaction and impulsivity. These negative effects may be also mediated by a partial sleep deprivation that can be related with high-perceived stress amplified by quarantine conditions (Zhao et al., 2020). It is possible that intrusive thoughts of guilt may elicit a dysfunctional mechanism that results in poor sleep quality and lower emotional competence (Brand et al., 2016).

Feelings of guilt may also be amplified by social media exposure during quarantine that can have a negative role on individual psychological well-being. Recent data among Chinese citizens analyzed by Gao et al. (2020) evidenced that frequently social media exposure is associated with high prevalence of mental health problems during the COVID-19 outbreak. Social media may lead to misinformation overload that can amplify feelings of personal hyper-responsibility elicited by guilt during quarantine period (World Health Organization., 2020).

Maladaptive guilt may emerge among health care workers operating in the “red zones” of COVID-19 intensive units. Given that in some countries, intensive care resources are limited, hospital doctors may be forced to choose whose life to save, necessitating difficult decisions about whom to treat first (Greenberg et al., 2020). Health care workers may be forced to exhausting work shifts with no time to rest and a few hours of sleep per day. As recently pointed out, this condition can lead to alterations in emotional reactivity (Tempesta et al., 2020) and to an increased risk propensity that can critically influence the decision process (Salfi et al., 2020). These experiences can be emotionally overwhelming, and guilt may be elicited by regret or remorse related to the decisions taken in critical conditions. Moreover, even staff members experienced in breaking bad news to relatives may be overcome by having to do this many times a day for weeks, especially where they have genuine feelings of guilt (Greenberg et al., 2020).

In all the situations presented above, both moral injury and burnout may affect mental health. Oppressive guilt may have disruptive effects ranging from acute stress responses to somatization as well as acute psychological trauma symptoms (Tangney and Dearing, 2002). As previously noted, people may be paralyzed by re-experiencing negative situations related to past mistakes and may exhibit avoidant behaviors toward contexts where the perceived risk of possible guilt remains high.

## The Shame of COVID-19

As demonstrated in previous pandemics (HIV, Hepatitis B, Ebola), public health and social responses to COVID-19 have the potential to exacerbate stigma and shameful experiences that can be dangerous (Logie and Turan, 2020).

Tangney and Dearing evidenced that the emotion of shame induces toxic experiences of worthlessness, inferiority, and incompetence and leads to a desire to escape and socially withdraw (Tangney and Dearing, 2002; Tracy et al., 2007). Recursive experiences of shame can exacerbate a global negative self-attribution that is often associated with adverse effects on mental well-being such as poor psychological adjustment, interpersonal difficulties, and overall poor life functioning (Cavalera et al., 2018; Pietrabissa et al., 2018). Psychopathological symptoms elicited by shameful experiences can vary from eating disorders and depression to anxiety disorders (Cândea and Szentagotai-Tǎta, 2018; Oliveira et al., 2018; Melo et al., 2020). The highly distressing and cumulative nature of COVID-19-related stressors can often be an ideal situation to result in shame response (although the actual prevalence will not be known for some time) (Haller et al., 2020; Ransing et al., 2020). Therefore, shame related to COVID-19 can engender traumatic aspects based on perceptions of having no value to others, or worse, of being dangerous to them (Dorahy et al., 2017). The fear of possible shameful experiences and of being rejected by others can prevent individuals from disclosing relevant facts about their clinical situation and their actual risk of contagion (Taylor, 2001). Such an attitude may be found in traumatized patients treated for COVID-19 or who are showing possible symptoms of the disease and can potentially lead to the subsequent spread of the virus in other social contexts.

Personal experiences of COVID-19 may elicit shame related to thoughts of inferiority and weakness. After getting infected, people may perceive themselves as defective and powerless, triggering self-criticism toward the entire self. Moreover, COVID-19 patients or even people who are no longer infected with the disease may manifest shameful feelings induced by social rejection by other family members or friends (Brooks et al., 2020). Given that shame can make people want to withdraw from the world for longer periods than recommended by doctors, the task of living with COVID-19 may represent a far more negative experience than it should be or needs to be.

The shame of infecting others for COVID-19 patients is a key element to handle and understand during admission/ward stay and during quarantine (Sahoo et al., 2020). Narrative lived experiences of patients with COVID-19 evidenced shameful intrusive thoughts and the fear of being stigmatized by friends, colleagues, and neighbors (Sahoo et al., 2020). These negative feelings can be amplified by social media exposure that is characterized by an abundance of misinformation and fake news circulating about COVID-19 (Islam et al., 2020). This unpleasant emotion can be amplified by social disparities as well as by the spread of stigma associated with COVID-19, affecting not only those newly diagnosed with the disease but also health care providers. As in many cases, the source of COVID-19 cannot be easily traced, and social stigma may arise toward people who have the most contact with patients (Galbraith et al., 2020). Thus, nurses, doctors, and health care providers as a whole may be perceived by other people as “unsafe” because of their job and thereby become victims of avoidant behaviors.

Moreover, as shame elicits strong self-deprecating reactions of the entire self, it may trigger self-aggressive and suicidal behaviors (Tangney and Dearing, 2002). The relationship between traumatic life events and self-aggressive behaviors is well documented and trauma from disaster events can increase suicidal ideation among laypeople and emergency workers alike (Galbraith et al., 2020). As evidenced for guilt, also in this case, it's important to pay attention to quality of life conditions related to quarantine. When long lockdown situations are combined with sleep loss, this situation can strongly affect emotional reactivity (Brand et al., 2016; Salfi et al., 2020), increasing negative affective tone and negative judgments related to positive stimuli (Tempesta et al., 2020). As the first cases on suicides related to COVID-19 are now receiving the attention of the scientific community (Thakur and Jain, 2020), social stigma and anticipatory shame are key factors that deserve to be explored.

## Immediate Psychological Interventions

Given the psychological implications of COVID-19, the development of specific treatment programmes for both health care staff and for the population as a whole is becoming a priority (Logie and Turan, 2020). World Health Organization mentions that psychological issues need to be taken into considerations during the COVID-19 pandemic for the general public (World Health Organization., 2020). Many authors have also stressed the psychological first aid to be provided to the patients admitted in the COVID wards (Bo et al., 2020; Wang et al., 2020). Within this perspective, special efforts should be made to tackle shameful stigmas and guilty maladaptive feelings as they can be strongly related with severe mental health complications (World Health Organization., 2020). This can be achieved by adopting specific therapeutic approaches that help people to develop positive perceptions of themselves or to engage in reparative behaviors (Molinari and Cavaleri, 2015). Compassion-focused therapy may represent useful interventions in managing shameful negative thoughts and providing care and kindness (Gilbert, 2014). Hyper-responsibility and maladaptive shame may be managed through groups' interventions or specified individual treatments (McGarty et al., 2005; Dearing and Tangney, 2011). Where the shame and guilt elicited by COVID-19 are related to negative traumatic symptoms or overlap with past traumas, eye movement desensitization reprocessing intervention is also a relevant option (Shapiro, 2017).

As COVID-19's negative psychological implications can be found among doctors irrespective of whether they work directly with infected patients, special attention to health care providers is required (Greenberg et al., 2020). Given the pressure to maintain high-quality health care provision during a pandemic, it is possible that this kind of professional commitment may relate strongly to psychological distress. As doctors' reluctance to seek help or to disclose their difficulties has been previously shown, such behaviors may be amplified by experiences of guilt and shame (Galbraith et al., 2020).

Finally, from a social point of view, the people most affected by COVID-19 can be engaged in developing stigma mitigation strategies, although they may experience social and health disparities.

The risk of disinformation may help facilitate stigma and xenophobia by reproducing the social construction of the illness as a foreign invasion, in turn reinforcing social hierarchies and power inequities (Logie and Turan, 2020). Conversely, lived experiences of COVID-19 and other intersecting stigmas may inform contextually specific and stigma-informed public health approaches. Therefore, social media intelligence should be harnessed to enhance the needed mobilization of the public and local communities to follow quarantine procedures, quickly decrease the spread of fears and uncertainty, and enhance public trust in public health measures (Depoux et al., 2020). Only by promoting collaborations with concerned communities and by providing smart guidance for public participation the efficacy of quarantine orders during emerging pandemic can be ensured.

## Author Contributions

CC drafted the manuscript, revised it critically, and finally approved it.

## Conflict of Interest

The author declares that the research was conducted in the absence of any commercial or financial relationships that could be construed as a potential conflict of interest.
